# Pathways from maternal depression to child resilience: Socioeconomic, family, and individual factors in the 2004 Pelotas (Brazil) birth cohort

**DOI:** 10.1002/jcv2.12188

**Published:** 2023-10-03

**Authors:** Jessica Mayumi Maruyama, Andreas Bauer, Gemma Hammerton, Sarah L. Halligan, Ina S. Santos, Tiago N. Munhoz, Aluísio J. D. Barros, Fernando C. Barros, Graeme Fairchild, Alicia Matijasevich

**Affiliations:** ^1^ Departamento de Medicina Preventiva Faculdade de Medicina FMUSP Universidade de São Paulo São Paulo Brazil; ^2^ Department of Psychology University of Bath Bath UK; ^3^ Human Development and Violence Research Centre (DOVE) Federal University of Pelotas Pelotas Brazil; ^4^ Postgraduate Program in Epidemiology Federal University of Pelotas Pelotas Brazil; ^5^ Population Health Sciences Bristol Medical School University of Bristol Bristol UK; ^6^ MRC Integrative Epidemiology Unit University of Bristol Bristol UK; ^7^ Department of Psychiatry and Mental Health University of Cape Town Cape Town South Africa; ^8^ Postgraduate Program in Paediatrics and Child Health Pontifical Catholic University of Rio Grande do Sul Porto Alegre Brazil; ^9^ Faculty of Psychology Federal University of Pelotas Pelotas Brazil; ^10^ Postgraduate Program in Health and Behaviour Catholic University of Pelotas Pelotas Brazil

**Keywords:** Cognitive stimulation, IQ, maternal depression, mediation, resilience, socioeconomic status

## Abstract

**Background:**

The negative impacts of maternal depression on child mental health outcomes are well‐documented. However, some children show adaptive functioning following exposure to maternal depression, demonstrating resilience. In a large birth cohort from Brazil, a middle‐income country, we examined direct and indirect pathways, considering socioeconomic, family, and individual factors, contributing to the development of resilience.

**Methods:**

Using data from the 2004 Pelotas Birth Cohort (*N* = 4231), we restricted the sample to those exposed to maternal depression up to age 6 years (depression present at ≥2 out of 5 assessment waves; *n* = 1132; 50% boys). Resilience was defined as scoring below or equal to the mean of the unexposed group on all four problem subscales of the parent‐report Strengths and Difficulties Questionnaire at age 11 years. We examined pathways from socioeconomic status (SES; measured at birth) to resilience via cognitive stimulation (CS) (at 24 and 48 months) and Intelligence quotient (IQ) (at 6 years), and from CS to resilience via IQ, using counterfactual mediation.

**Results:**

A minority of children exposed to maternal depression showed resilience (12.4%). There was evidence of indirect pathways from SES to resilience via CS (odds ratio (OR) = 1.76, 95% confidence interval (CI) 1.02–3.38) and IQ (OR = 1.19, 95% CI 1.01–1.42), such that higher SES was associated with resilience via both higher levels of CS and higher IQ, which, in turn, were each positively associated with resilience. Furthermore, there was evidence of a direct (OR = 1.86, 95% CI 1.01–3.76) and total effect (OR = 1.94, 95% CI 1.05–3.89) of CS on resilience, even after controlling for SES. However, these effects varied depending on how persistent and severe depression was defined.

**Conclusions:**

These findings suggest that CS in early childhood may represent a modifiable protective factor for children exposed to maternal depression and a promising intervention target to promote child resilience in the context of maternal depression exposure.

## Graphical abstract

In a large Brazilian birth cohort, we identified just 12.4% of children exposed to maternal depression who were free of psychopathology. Higher SES was associated with resilience indirectly via both higher levels of cognitive stimulation and higher IQ. Cognitive stimulation was associated with resilience irrespective of SES. Child cognitive stimulation may represent a modfiiable resilience factor in children exposed to maternal depression.

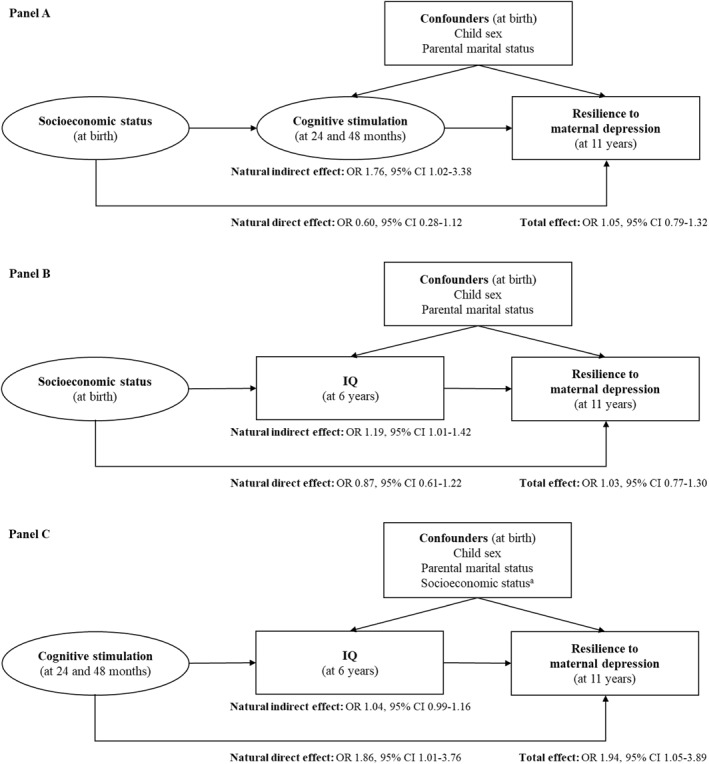

**Key points**

**Some children show an ability to cope with and adapt to maternal depression, demonstrating resilience.**

- Socioeconomic, family, and individual factors have been identified as predictors of resilience in at‐risk children, but the pathways linking these variables are poorly understood.
- Just 12.4% of the children exposed to maternal depression were free of psychopathology as assessed using the Strengths and Difficulties Questionnaire (SDQ).
- Higher SES was associated with resilience indirectly via both higher levels of cognitive stimulation (CS) and higher IQ; additionally, children who received higher levels of CS were more likely to be resilient, irrespective of their SES.
- Child CS may represent a modifiable resilience factor, especially in at‐risk families, and increasing CS may be effective in promoting resilience in children exposed to maternal depression.

## INTRODUCTION

Maternal depression is a highly prevalent psychiatric disorder, representing a major public health concern (Hahn‐Holbrook et al., 2017), and has been shown to be strongly linked to offspring psychopathology (Meaney, 2018; Slomian et al., 2019). However, some children show an ability to cope with and adapt to maternal depressive symptoms, demonstrating resilience (Collishaw et al., 2016; Mahedy et al., 2018). Resilience can be defined as a dynamic ability to maintain psychological health in the face of adversity (Masten & Powell, 2003; Sisto et al., 2019); consequently, it cannot be assessed directly but must be inferred from other measures, resulting in a variety of definitions and approaches across studies (Cosco et al., 2017). For example, the person‐centred method compares individuals exposed to similar types of adversity to determine what differentiates those who remain well from those who develop mental health problems (Cosco et al., 2017; Masten & Powell, 2003). A thorough understanding of how resilience develops is crucial in identifying intervention targets to improve child outcomes related to maternal depression.

Previous research has identified factors at multiple levels that may protect children from the harmful effects of maternal depression. These include: (i) *socioeconomic factors*: family income, maternal education, and neighborhood quality (Giallo et al., 2018; Masten & Powell, 2003); (ii) *family factors*: paternal emotional support, maternal warmth, and low marital conflict (Mahedy et al., 2018); and (iii) *individual factors*: child self‐esteem, self‐efficacy, and intelligence quotient (IQ) (Collishaw et al., 2016). Importantly, these factors are likely to co‐occur and/or may share common pathways in the development of resilience (Collishaw et al., 2016). For example, parents from higher SES backgrounds may provide a more enriching and stimulating environment for their children. Indeed, early CS, such as being read to early in life, has been shown to improve cognitive functioning (Byford et al., 2012; Gartland et al., 2019), which, in turn, has been associated with resilience in children exposed to maternal depression (Khambati et al., 2018; Lewandowski et al., 2014; Pargas et al., 2010). This may be due to improved coping skills, such as enhanced self‐regulation, as a result of higher cognitive development (Pargas et al., 2010). Thus, SES may influence child resilience not directly but through indirect pathways, such as increased CS, which may result in greater cognitive abilities to buffer against exposure to maternal depression (Giallo et al., 2018).

To date, the majority of studies examined these factors individually as opposed to collectively, largely ignoring their interrelatedness, which may have led to faulty conclusions when assessing the importance and role of specific resilience factors. Furthermore, previous research has been largely limited to cross‐sectional designs, which complicates our understanding of the directionality and causality of these associations, and smaller scale longitudinal studies, with limited generalisability to broader populations (Cosco et al., 2017). Related to the latter point, the vast majority of studies have been conducted in high‐income countries, and little is known regarding whether findings translate to low‐ and middle‐income countries. Although we would expect that children from around the world will benefit from a stimulating environment, demonstrating this is critical to developing interventions that reflect the full range of social and cultural contexts in which children grow and develop (Herba et al., 2016).

To address these gaps in the literature, we used prospective longitudinal data from a large birth cohort from Brazil, a middle‐income country, to examine direct and indirect pathways contributing to the development of resilience in 11‐year‐old children exposed to maternal depression in early life. We considered socioeconomic, family, and individual factors that have previously been associated with resilience, including SES, CS, and IQ. We hypothesized that higher SES will exert (i) direct positive effects on child resilience, and (ii) indirect effects by being positively associated with higher levels of child CS and IQ scores. Furthermore, we hypothesized that (iii) CS will predict increased resilience via direct and IQ‐mediated indirect pathways, even after controlling for the effect of SES. To test the robustness of our findings, we also examined two additional approaches to define exposure to maternal depression based on the duration and severity of depressive symptoms.

## METHODS

### Participants

The 2004 Pelotas Birth Cohort is an ongoing population‐based, prospective longitudinal study located in Pelotas (Rio Grande do Sul), a southern Brazilian city. Out of 4263 live births, 4231 children (99.2%) were included. The births were identified through daily visits to the five maternity hospitals in the city, where >98% of all city deliveries took place. Mothers and their children were assessed at birth, 3, 12, 24, and 48 months, and 6 and 11 years. Follow‐up rates ranged from 86.6% to 99.2%. The current study focused on a subsample of children exposed to maternal depression as defined below (*n* = 1132), with the remaining unexposed children (*n* = 2430) being excluded. Further details on the 2004 Pelotas Birth Cohort can be found elsewhere (Santos et al., 2011, 2014).

### Ethical considerations

All assessments were approved by the Federal University of Pelotas Medical School Research Ethics Committee. Mothers were informed of all follow‐up procedures, the study objectives, the voluntary nature of their participation, and their right not to participate, to answer specific questions, and to the confidentiality of their information.

### Measures

#### Socioeconomic status (SES)

SES was assessed at birth by maternal self‐report, using three continuous variables: (i) family income in the month prior to the child's birth, measured in Brazilian Real (BRL), with 2.89 BRL equating approximately to 1 USD in 2004 when recruitment of the families commenced; and (ii) maternal and (iii) paternal education, coded as complete school years of formal education.

#### Child cognitive stimulation

At ages 24 and 48 months, mothers were asked three questions related to child CS in the past week, which have been used in previous studies (Barros et al., 2010). The three items asked whether the child: (i) was read to or told a story; (ii) went to a park or playground; and (iii) had a children's book at home. All items were coded dichotomously.

#### Intelligence quotient

At age 6 years, children completed a short form of the Wechsler Intelligence Scale for Children‐III (WISC‐III; Wechsler, 2002). The short‐form WISC‐III was composed of two verbal (*similarities* and *arithmetic*) and two performance (*block design* and *picture completion*) subtests and has been shown to correlate highly (*r* = 0.94) with full‐scale IQ in children aged 6 years (Kaufman et al., 1996).

#### Child resilience to maternal depression

Maternal depression was assessed at 3, 12, 24, 48 months, and 6 years, using the self‐reported Edinburgh Postnatal Depression Scale (EPDS) (Cox et al., 1987). The 10 items are each rated on a 4‐point scale (0–3), yielding total scores ranging from 0 to 30. Following a validation study of the EPDS in the same sample, we used a cut‐off score of ≥10 (Santos et al., 2007).

In the main analysis, the current sample was limited to those exposed to maternal depression as defined by depression being present at ≥2 assessment waves (*exposed group*, *n* = 1132; 50% boys); all other children were assigned to the *unexposed group* and excluded from subsequent analyses (*n* = 2430; 52% boys).

The parent‐reported SDQ was used to assess child functioning and psychopathology at age 11 years (Goodman et al., 2000). We used four of the subscales, including emotional problems (e.g., “Many worries, often seems worried”), conduct problems (e.g., “Often fights with other children or bullies them”), peer problems (e.g., “Picked on or bullied by other children”), and hyperactivity (e.g., “Restless, overactive, cannot stay still for long”). Each subscale consists of five items, which are rated on a 3‐point scale (from *Not true* = 0 to *Certainly true* = 2), yielding scores from 0 to 10.

Children in the exposed group who scored below or equal to the mean of the unexposed group on all four SDQ subscales were classified as “resilient” (*n* = 139, 12.3% of the exposed group), while *n* = 933 were classified as “non‐resilient”. To test the robustness of our findings, we used two additional approaches to define exposure to maternal depression: (i) *longer duration*, that is, maternal depression being present at ≥3 assessment waves with EPDS scores of ≥10 (*n* = 637), with 70 (11.0%) children being classified as resilient in this case; and (ii) *greater severity*, that is, EPDS scores of ≥13 being present at ≥2 assessment waves (*n* = 592), with 59 (10.0%) children being classified as resilient in this instance.

#### Confounders

All models were adjusted for child sex (*male* or *female*) and parental marital status (*married/living with a partner* or *single/living alone*).

### Analysis strategy

All analyses were performed in Mplus, Version 8.1 (Muthén & Muthén, 2017). We derived two latent factors, including SES with three indicators (family income as well as maternal and paternal education) and CS (at ages 24 and 48 months) with six indicators. This measurement model was re‐specified as a confirmatory factor analysis model with correlated factors, and assessed with the following model fit indices: Comparative Fit Index (CFI) and Tucker‐Lewis Index (TLI), Root Mean Square Error of Approximation (RMSEA), and Standardised Root Mean Residual (SRMR), with values of ≥0.95, ≤0.06, and ≤0.08, respectively, indicating good model fit (Hu & Bentler, 1999).

**FIGURE 1:**
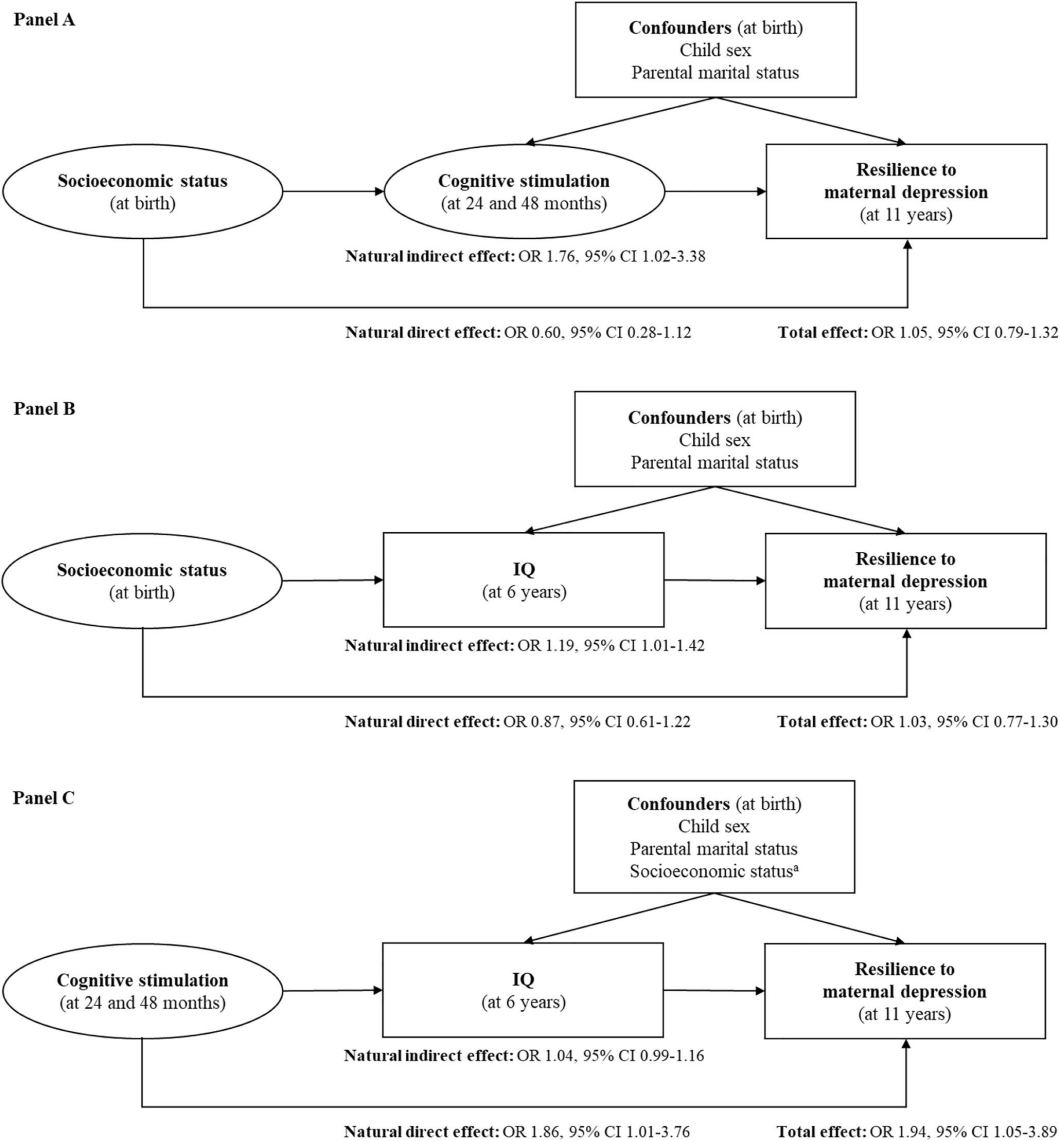
Single‐mediator models representing the hypotheses that cognitive stimulation (CS) is a mediator of the association between socioeconomic status and resilience to maternal depression (Panel A) and Intelligence quotient (IQ) is a mediator of the association of socioeconomic status and CS with resilience (Panels B and C), using counterfactual mediation and after adjusting for confounders. *Note*. Effect definitions for counterfactual mediation are provided in Table S1. Latent variables are presented as ellipses. Observed variables are presented as rectangles. A Latent variable.

Counterfactual mediation is recommended when using common (>10%) binary outcomes (VanderWeele, 2015), which was the case across approaches on how persistent and severe depression was defined. However, this meant that it was not possible to examine the effects of multiple mediators sequentially, as this method is not well‐established for counterfactual mediation with latent factors. More specifically, the indirect effect from SES to CS to IQ and then to resilience could not be estimated. Thus, we estimated a series of single‐mediator models representing the hypotheses that: (i) CS is a mediator of the association between SES and resilience; (ii) IQ is a mediator of the association between SES and resilience; and (iii) IQ is a mediator of the association between CS and resilience (Panels A‐C in Figure 1 represent schematic diagrams for each mediation model). We assessed the *natural direct effect*, *natural indirect effect*, and *total effect* (see Table S2, available online, for effect definitions in counterfactual mediation).

All models were adjusted for child sex and parental marital status; the models examining CS as an exposure variable were additionally adjusted for SES. There were small amounts of missingness for family income, maternal education, and CS (≤2%), whereas IQ (5.9%) and paternal education (22.2%) showed higher rates of missingness. Results are presented as odds ratios (ORs) with 95% confidence intervals (CIs), which are expressed in standardised units (i.e., per increase in standard deviation), and represent conditional effects for those with a score of zero on confounders (i.e., boys with mothers who are married or live with a partner). Odds ratios in the mediation models were calculated based on predicted probabilities from probit regression analyses using the “model indirect” command in Mplus with 1000 bootstrap samples (Muthén & Asparouhov, 2014).

In the main analysis, we focused on children exposed to maternal depression (defined as an EPDS score ≥10 at two or more time points); we subsequently examined whether the results replicate when using two alternative approaches to define exposure to maternal depression based either on a longer duration (defined as an EPDS score ≥10 at 3+ time points) or greater severity (defined as an EPDS score ≥13 at 2+ time points).

## RESULTS

### Descriptive statistics

Table 1 presents descriptive statistics for those included in the main analysis (exposed group) and those excluded (unexposed group). Children exposed to maternal depression had higher scores on all four SDQ subscales (Cohen's *d*s ranging between 0.46 and 0.52, all *p*s < 0.001). Depressed mothers reported lower monthly family income and parental education than mothers in the unexposed group, with small to medium effect sizes (*d*s ranging between 0.32 and 0.50, all *p*s < 0.001). Similarly, depressed mothers reported that their children received lower levels of CS at 24 and 48 months than mothers in the unexposed group, with ORs ranging from 0.41 to 0.77, all *p*s < 0.05. Finally, children exposed to maternal depression had a mean IQ 5.2 points lower than unexposed children (*d* = 0.34, *p* < 0.001), and were more likely to live with mothers who were single, divorced, or widowed (OR 1.31, 95% CI 1.08–1.59). Correlations between all study variables are presented in the supplement (Figure S1).

**TABLE 1:** Descriptive statistics for the total sample and those exposed versus unexposed to maternal depression up to age 6 years.

	Total sample (*N* = 3562)	Maternal depression		Cohen's *d* (*p*‐value) or OR (95% CI)
		Unexposed (*n* = 2430)	Exposed (*n* = 1132)	
	Mean (SD) or *n* (%)	Mean (SD) or *n* %	Mean (SD) or *n* %	
Continuous variables				
SDQ				
Conduct problems (0–10)	1.4 (1.8)	1.1 (1.6)	2.0 (2.1)	0.52, *p* < 0.001
Emotional problems (0–10)	2.7 (2.3)	2.3 (2.1)	3.4 (2.6)	0.48, *p* < 0.001
Hyperactivity (0–10)	3.2 (3.0)	2.8 (2.8)	4.1 (3.1)	0.46, *p* < 0.001
Peer problems (0–10)	1.4 (1.7)	1.1 (1.6)	1.9 (2.0)	0.46, *p* < 0.001
Socioeconomic status				
Monthly family income (BRL)	802.4 (1096.0)	911.9 (1164.4)	567.9 (889.0)	0.32, *p* < 0.001
Maternal education (years)	8.2 (3.4)	8.7 (3.4)	7.0 (3.1)	0.50, *p* < 0.001
Paternal education (years)	7.9 (3.7)	8.4 (3.7)	7.0 (3.4)	0.37, *p* < 0.001
IQ (40–160)	96.8 (15.4)	98.5 (15.6)	93.3 (14.4)	0.34, *p* < 0.001
Categorical variables				
Cognitive stimulation (0–3)				
At 24 months		*p* < 0.001		
0	627 (18.3)	368 (15.8)	259 (23.4)	Ref
1	1025 (29.9)	666 (28.6)	359 (32.4)	0.77 (0.62–0.94)
2	1185 (34.5)	833 (35.8)	352 (31.7)	0.60 (0.49–0.73)
3	597 (17.4)	458 (19.7)	139 (12.5)	0.43 (0.34–0.55)
At 48 months		*p* < 0.001		
0	257 (7.5)	136 (5.9)	121 (10.9)	Ref
1	712 (20.8)	432 (18.6)	280 (25.2)	0.73 (0.55–0.97)
2	1309 (38.2)	908 (39.2)	401 (36.1)	0.50 (0.38–0.65)
3	1152 (33.6)	843 (36.4)	309 (27.8)	0.41 (0.31–0.54)
Binary variables				
Child sex (female)	1728 (48.5)	1162 (47.8)	566 (50.0)	1.09 (0.95–1.26)
Maternal relationship status (single, divorced, or windowed)	556 (15.5)	351 (14.4)	205 (18.1)	1.31 (1.08–1.59)

*Note*: Based on listwise deletion. Observed, rather than latent, variables are presented.

Abbreviations: BRL, Brazilian Real (2.89 BRL = 1 USD in January 2004 when recruitment of the families commenced); CI, Confidence interval; OR, Odds ratio; SDQ, Strengths and Difficulties Questionnaire.

The measurement models, factor loadings and model fit of the three approaches based on the levels of exposure to maternal depression are presented in Table S1 (see online supplement). All standardized factor loadings were significant at *p* < 0.01. In the main analysis, the factor loadings (standard error) varied from 0.445 (0.014) to 0.845 (0.033) for the socioeconomic status (SES) factor, and from 0.297 (0.025) to 0.923 (0.040) for the CS factor. The SES and CS factors were strongly positively correlated (*r* = 0.724, S.E. = 0.033, *p* < 0.001). The model fit for the main analysis was only modest: CFI/TLI = 0.868/0.817, RMSEA (90% CI) = 0.127 (0.117–0.137), SRMR = 0.104; the measurement models using the other two approaches to defining maternal depression showed good model fit.

### Pathways from maternal depression to child resilience

First, we examined CS as a mediator of the association between SES and resilience (see Panel A in Figure 1). There was no evidence of a direct effect from SES on resilience (OR 0.60, 95% CI 0.28–1.12). However, there was evidence of an indirect effect of SES on resilience via CS (OR 1.76, 95% CI 1.02–3.38). More specifically, higher SES was associated with resilience indirectly via higher levels of CS. Finally, there was no evidence of a total effect of SES on resilience (OR 1.05, 95% CI 0.79–1.32).

Next, we examined IQ as a mediator of the association between SES and resilience (see Panel B in Figure 1). Again, there was no evidence of a direct effect of SES on resilience (OR 0.87, 95% CI 0.61–1.22). However, there was evidence of an IQ‐mediated indirect effect from SES on resilience (OR 1.19, 95% CI 1.01–1.42). More specifically, higher SES was associated with resilience via higher levels of IQ. Again, there was no evidence of a total effect (OR 1.03, 95% CI 0.77–1.30).

Finally, we examined IQ as a mediator of the association between CS and resilience (see Panel C in Figure 1). We found evidence of a direct effect of CS on resilience (OR 1.86, 95% CI 1.01–3.76). In contrast, there was no evidence of an IQ‐mediated effect from CS to resilience via IQ (OR 1.04, 95% CI 0.99–1.16). Finally, there was evidence of a total effect of CS on resilience (OR 1.94, 95% CI 1.05–3.89).

In sum, we identified indirect pathways from SES to resilience via CS and IQ. Additionally, there was evidence for direct and total effects of CS on resilience, even when adjusting for SES.

When re‐running these analyses defining exposure to maternal depression based on a *longer* duration (i.e., 3 rather than 2 instances of maternal depression over the measurement period), only the indirect effect from SES to resilience via IQ was replicated (OR 1.20, 95% CI 1.01–1.67). By contrast, there was no evidence of any indirect, direct, or total effects when defining exposure to maternal depression based on a greater severity (i.e., an EPDS cut‐off of 13 rather than 10). See Table 2 for more details.

**TABLE 2:** Results from counterfactual mediation models using alternative definitions of resilience.

	OR (95% CI)
EPDS ≥ 10 at 3+ time points (*N* = 597)	
Resilience defined as a low score on all four SDQ subscales	
SES → cognitive stimulation → resilience	
Natural indirect effect	1.47 (0.69–3.38)
Natural direct effect	0.67 (0.26–1.61)
Total effect	0.98 (0.67–1.38)
SES → IQ → resilience	
Natural indirect effect	**1.29 (1.01–1.67)**
Natural direct effect	0.76 (0.43–1.20)
Total effect	0.98 (0.67–1.38)
Cognitive stimulation → IQ → resilience	
Natural indirect effect	1.06 (0.97–1.27)
Natural direct effect	1.46 (0.54–3.31)
Total effect	1.55 (0.58–3.60)
EPDS ≥ 13 at 2+ time points (*N* = 558)	
Resilience defined as a low score on all four SDQ subscales	
SES → cognitive stimulation → resilience	
Natural indirect effect	1.27 (0.50–3.52)
Natural direct effect	0.77 (0.26–2.39)
Total effect	0.99 (0.59–1.54)
SES → IQ → resilience	
Natural indirect effect	1.21 (0.90–1.64)
Natural direct effect	0.80 (0.45–1.50)
Total effect	0.97 (0.58–1.56)
Cognitive stimulation → IQ → resilience	
Natural indirect effect	1.05 (0.98–1.27)
Natural direct effect	1.27 (0.41–3.62)
Total effect	1.33 (0.45–3.86)

Abbreviations: EPDS, Edinburgh Postnatal Depression Scale; SDQ, Strengths and Difficulties Questionnaire.

Bold values indicate statistically significant associations at *p* < 0.05.

## DISCUSSION

The current study investigated socioeconomic‐, family‐, and individual‐level variables that may contribute to the development of resilience in 11‐year‐old children exposed to maternal depression in early life. The findings demonstrate that, depending on how exposure to maternal depression is defined, protective factors may act differently. In our main analysis defining maternal depression as an EPDS score of ≥10 at 2+ time points, we found indirect pathways from SES to resilience via CS and IQ. Additionally, children who received higher levels of CS were more likely to show resilience, even after controlling for the effects of SES, in the model examining IQ as a mediator. When we re‐examined these pathways by extending the duration of maternal depression (i.e., an EPDS score of ≥10 at 3+ time points), there was only evidence of an indirect effect from SES to resilience via IQ. By contrast, when focusing on a greater severity of maternal depression (i.e., an EPDS score of ≥13 at 2+ time points), neither SES nor IQ nor CS demonstrated protective effects.

In our main analysis, CS predicted child resilience to maternal depression regardless of SES. Early CS may contribute to the development of resilience to maternal depression as part of a broader dimension of positive parenting practices (Zimmerman et al., 2005). For example, Giallo et al. (2018) found that maternal involvement in cognitively stimulating activities was associated with resilience status in children exposed to maternal depression. The authors proposed that interactions between caregivers and their children can provide opportunities to create a responsive environment and strengthen the parent‐child bond. They further highlighted that CS may improve child cognitive functioning, which may be directly related to resilience outcomes, possibly as a result of improved coping skills. This latter explanation is also supported by findings of the current study, with CS showing both a total and a direct effect on resilience status. Taken together, the current study identified CS as a potential modifiable resilience factor. Thus, interventions targeting depressed mothers may include components that encourage them to engage in cognitively stimulating activities, which may result in benefits to their child through three possible pathways: (1) improvement of child cognitive functioning, which may be directly associated with resilience outcomes; (2) promotion of positive child‐caregiver interactions that may develop other non‐cognitive skills related to positive mental health; and (3) providing support for depressed mothers to engage in and maintain activities that promote parental warmth and a sensitive caregiving environment.

The CS scale used in the current study may be a proxy for material resources, such as having children's books at home. Low family income is the most commonly used measure to evaluate economic deprivation and usually co‐occurs with other adverse conditions, such as material hardship (Reiss, 2013). Ashiabi and O’Neal (2007) showed that although family income is independently associated with child mental health outcomes, it also exerts strong indirect effects through material hardship and related parental factors such as stress levels. In addition, Gershoff et al. (2007) found that low family income and material hardship together impacted child behavior through parent‐mediated pathways; they also reported that the decrease in parental stress due to an increased income was almost entirely the result of a reduction in material hardship. Considering these findings, low‐income families would benefit the most from child CS interventions as they are more vulnerable to stress caused by economic strain and may find themselves unable to provide enriching and stimulating materials to their children (Crosnoe et al., 2010; Gershoff et al., 2007). Focusing on low‐income children may be particularly crucial in low‐ and middle‐income countries, where inadequate CS was found to be a key risk factor preventing children from achieving their full potential (Walker et al., 2007).

To test the robustness of these findings, we used two additional approaches to define exposure to maternal depression based on either more persistent (i.e., 3+ time points) or more severe (i.e., an EPDS score ≥13) depressive symptoms. These findings showed that some of these factors may be protective even in the case of more *recurrent* episodes of maternal depression, especially the positive effect of SES on IQ, which, in turn, may lead to resilience. By contrast, in more severe cases of maternal depression, the investigated protective factors may not suffice to counteract the detrimental effects of maternal depressive symptoms. Thus, particularly in these cases, children may be at risk of showing poor mental health outcomes, despite some favourable environmental conditions. Taken together, we hypothesize that CS is associated with resilience only when maternal depressive symptoms are not severe or persistent enough to affect mothers' parenting behavior. It is expected that women with more severe depressive symptoms, in comparison to women with mild or no symptoms, would present less sensitive, responsive, and engaged parenting behavior. Related to this, Lovejoy et al. (2000) reported a small but significant detrimental effect of maternal depression on positive parenting practices, especially for women from socioeconomically disadvantaged backgrounds. In addition, Guyon‐Harris et al. (2022) found that individual differences in maternal depressive symptoms are associated with different dimensions of parenting. In contrast to the current study and Lovejoy and colleagues' findings, however, the authors also reported that positive parenting (as measured by the same CS scale as used in the present study) did not differ by maternal depression profile. More research on the heterogeneity of maternal depressive symptoms and their impact on parenting and child outcomes is needed to disentangle the contradictory findings among the studies. Moreover, it should also be noted that other researchers have argued that it is important to consider outcomes beyond psychopathology, such as academic performance or social functioning, when assessing resilience (Burt et al., 2016; DuMont et al., 2007).

Key strengths of the current study include the use of a prospective, longitudinal birth cohort, spanning from birth to age 11 years, with high retention rates. Furthermore, the longitudinal design strengthens confidence in our findings related to developmental pathways, as the mediating variables were assessed after the exposure, but before the outcome variables were measured. In addition, maternal depression was measured repeatedly across a child's early life, which enabled us to assess recurrent and severe depression as opposed to isolated depressive episodes. Moreover, in contrast to previous studies, we tested the robustness of our findings by using different approaches to categorise maternal depression. Finally, this is one of the few studies that examined pathways from maternal depression to child resilience in a middle‐income country, where risk factors for both maternal and child mental health problems, such as socioeconomic disadvantage, are much more frequently experienced and potentially more severe than in many high‐income settings (Herba et al., 2016).

However, several limitations of the study need to be considered. First, indirect effects from SES to resilience through IQ may be overestimated as we could not adjust for the effect of child CS, which represents an intermediate confounder (i.e., a confounder of the mediator‐outcome association which is on the causal pathway between the exposure and outcome). Methods for accounting for intermediate confounders are not well‐established using counterfactual mediation with latent variables. Second, the absence of a total effect from SES to resilience may be explained by the occurrence of inconsistent mediation (i.e., suppressor effect), which is present when the direct and indirect effects have opposite signs; the result is a smaller total effect when adding them together. Alternatively, the absence of a total effect in the presence of indirect effects could be due to a lack of power or the presence of unmeasured mediator‐outcome confounding (Loeys et al., 2014; MacKinnon et al., 2002). Related to this, when using alternative approaches on how maternal depression was defined, our sample size was almost halved, which may have caused a significant loss in statistical power. Thus, particularly these findings need to be interpreted with caution. Third, the measurement model in the main analysis showed inadequate model fit, potentially indicating that our CS measure may not fully capture this construct. Furthermore, this may also partially explain the different results across maternal depression definitions; for example, correlations between SES and CS were stronger in the models based on the alternative approaches. Nevertheless, the current findings require replication with different CS indicators with better psychometric properties. Finally, child functioning was assessed at age 11 years, which encompasses the early years of adolescence. This stage of human development is characterized by dramatic changes in the biological, physical, emotional, and social environment (Dahl et al., 2018). It is possible that the effects of the studied protective factors in predicting resilience would differ according to developmental stage. For instance, the impact of CS might be greater if the outcomes were measured at younger ages, such as middle childhood. In addition, we cannot tell whether the effects of the identified protective factors continue into later adolescence or adulthood. Therefore, future studies should consider investigating the impact of exposure to maternal depression at different stages of childhood and adolescence to investigate resilience across the lifespan.

## CONCLUSION

The findings from the current study suggest that SES may not directly influence resilience in children exposed to maternal depression, but rather through indirect pathways involving child CS and IQ. The importance of these mediating factors is further highlighted by the fact that children who experienced higher levels of CS in early childhood were more likely to be resilient, regardless of their socioeconomic background. Thus, early CS may represent a modifiable resilience factor against the harmful effects of maternal depression in children across the SES spectrum. However, these effects may vary depending on the duration and severity of exposure to maternal depression. Considering that just 12% of children were classified as resilient by virtue of remaining free of current mental health problems despite exposure to maternal depression, more research on the pathways from maternal depression to child resilience is urgently needed. The findings indicate that preventive interventions focusing on early CS, especially targeting at‐risk families, may represent a promising factor in promoting child mental health resilience.

## AUTHOR CONTRIBUTIONS

**Jessica Mayumi Maruyama**: Conceptualization; formal analysis; methodology; writing—original draft; writing—review and editing. **Andreas Bauer**: Conceptualization; formal analysis; methodology; writing—original draft; writing—review and editing. **Gemma Hammerton**: Conceptualization; formal analysis; methodology; supervision; writing—original draft; writing—review and editing. **Sarah L. Halligan**: Conceptualization; supervision; writing—original draft; writing—review and editing. **Ina S. Santos**: Data curation; funding acquisition; writing—review and editing. **Tiago N. Munhoz**: Data curation; funding acquisition; writing—review and editing. **Aluísio J. D. Barros**: Data curation; funding acquisition; writing—review and editing. **Fernando C. Barros**: Data curation; funding acquisition; writing—review and editing. **Graeme Fairchild**: Supervision; funding acquisition; writing—review and editing. **Alicia Matijasevich**: Supervision; funding acquisition; writing—review and editing.

## CONFLICT OF INTEREST STATEMENT

Graeme Fairchild is on the JCPP Advances Editorial Advisory Board. The remaining authors have declared that they have no competing or potential conflicts of interest.

## ETHICAL CONSIDERATIONS

All assessments were approved by the Federal University of Pelotas Medical School Research Ethics Committee. Mothers were informed of all follow‐up procedures, the study objectives, the voluntary nature of their participation, and their right not to participate, to answer specific questions, and to the confidentiality of their information.

## Supporting information

Supporting Information S1

## Data Availability

Applications to use the data can be made by contacting the researchers of the 2004 cohort (see http://www.epidemio‐ufpel.org.br/site/content/faculty/ for a list of key faculty members) and completing the application form (http://www.epidemio‐ufpel.org.br/site/content/studies/formularios.php). A list of administered questionnaires at each timepoint can be accessed online (http://www.epidemio‐ufpel.org.br/site/content/coorte_2004‐en/questionnaires.php). Researchers with successful applications will receive a dataset including the requested variables and unique participant IDs.
