# Supplementary material for: Pathways from maternal depression to child resilience: Socioeconomic, family, and individual factors in the 2004 Pelotas (Brazil) birth cohort
Source: JCPP Adv. 2023 Oct 3;3(4):e12188. doi: 10.1002/jcv2.12188 (PMC10694539; doi:10.1002/jcv2.12188)
Supplement: Supplementary file 1 — Supporting Information S1 [file JCV2-3-e12188-s001.docx]

**Supporting Information**

Pathways from maternal depression to child resilience: Socioeconomic, family, and individual factors in the 2004 Pelotas (Brazil) Birth Cohort, by Maruyama Bauer *et al.*

**Figure S1.** Correlation matrix of all study variables

**Table S1.** Measurement models specified as confirmatory factor analysis models with correlated factors based on varying levels of exposure to maternal depression

**Table S2.** Effect definitions for counterfactual mediation

**Figure S1.** Correlation matrix of all study variables


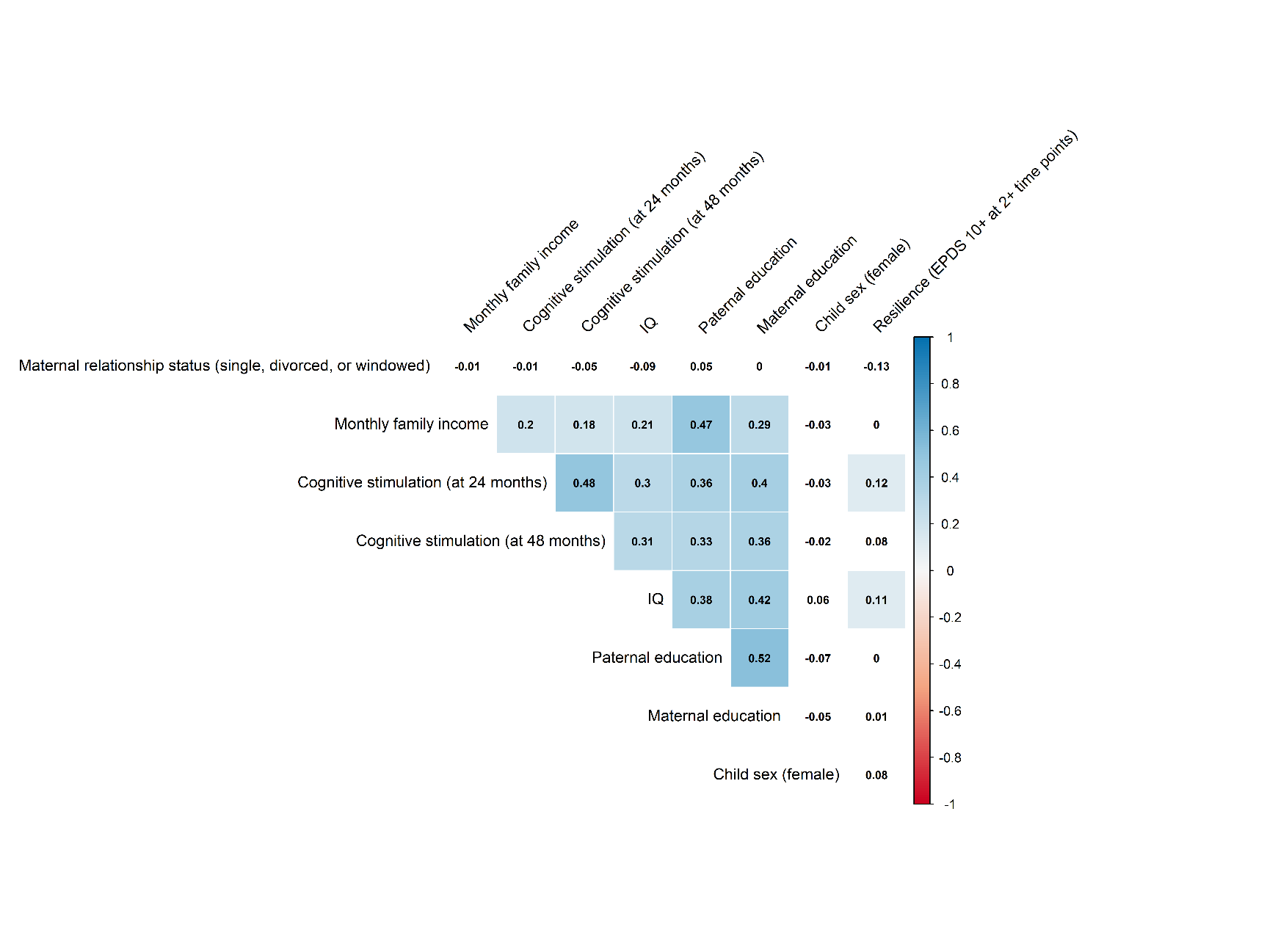


| **Table S1.** Measurement models specified as confirmatory factor analysis models with correlated factors based on varying levels of exposure to maternal depression | | | |
| --- | --- | --- | --- |
|  | **EPDS ≥ 10 in 2+ time points**  **(*N* = 1065)** | **EPDS ≥ 10 in 3+ time points**  **(*N* = 597)** | **EPDS ≥ 13 in 2+ time points**  **(*N* = 558)** |
|  | Factor loadings (S.E.) | Factor loadings (S.E.) | Factor loadings (S.E.) |
| **Socioeconomic status (SES) factor** |  |  |  |
| Monthly family income | 0.445 (0.014) | 0.598 (0.031) | 0.613 (0.034) |
| Maternal education | 0.845 (0.033) | 0.605 (0.031) | 0.611 (0.031) |
| Paternal education | 0.660 (0.020) | 0.690 (0.033) | 0.690 (0.034) |
| **Cognitive stimulation (CS) factor** |  |  |  |
| Was read to or told a story (24 months) | 0.346 (0.024) | 0.492 (0.052) | 0.533 (0.053) |
| Went to a park or playground (24 months) | 0.338 (0.046) | 0.286 (0.059) | 0.341 (0.059) |
| Had a children’s book at home (24 months) | 0.666 (0.038) | 0.668 (0.045) | 0.643 (0.046) |
| Was read to or told a story (48 months) | 0.297 (0.025) | 0.489 (0.054) | 0.486 (0.055) |
| Went to a park or playground (48 months) | 0.311 (0.045) | 0.359 (0.058) | 0.352 (0.059) |
| Had a children’s book at home (48 months) | 0.923 (0.040) | 0.791 (0.041) | 0.827 (0.042) |
|  |  |  |  |
| **Correlation between SES and CS factors** | *r* = 0.724 (0.033), *p* < 0.001 | *r* = 0.859 (0.050), *p* < 0.001 | *r* = 0.857 (0.052), *p* < 0.001 |
|  |  |  |  |
| **Model fit indices** |  |  |  |
| Comparative Fit Index (CFI) | 0.868 | 0.963 | 0.948 |
| Tucker-Lewis Index (TLI) | 0.817 | 0.949 | 0.928 |
| Root Mean Square Error of Approximation (RMSEA) | 0.127 | 0.052 | 0.065 |
| RMSEA 90% Confidence Interval (90%CI) | 0.117 – 0.137 | 0.037 – 0.068 | 0.050 – 0.080 |
| Standardised Root Mean Residual (SRMR) | 0.104 | 0.055 | 0.065 |
| ***Note.*** Standard errors (S.E.) are shown in parenthesis. All standardized factor loadings are significant at a *p* < 0.001. | | | |

| **Table S2.** Effect definitions for counterfactual mediation | |
| --- | --- |
| **Natural direct effect**  *Y_i_*(*a*,*M_i_*(a)) to  *Y_i_*(*a**,*M_i_*(a)) | The effect of changing the exposure from the exposure level of interest *a* (e.g., the population mean) to the comparison level *a** (e.g., 1 standard deviation above the population mean), while fixing the mediator *M* to the level it would take under the exposure level of interest *a*. |
| **Natural indirect effect** *Y_i_*(*a**,*M_i_*(a)) to *Y_i_*(*a**,*M_i_*(a*)) | The effect of changing the mediator *M* to the level it would take in response to changing the exposure from the exposure level of interest *a* (e.g., the population mean) to the comparison level *a** (e.g., 1 standard deviation above the population mean), while fixing the exposure to the comparison level *a**. |
| **Total effect** | Natural direct effect + natural indirect effect |
